# Allogeneic Red Blood Cell Transfusion Rate and Risk Factors After Hemiarthroplasty in Elderly Patients With Femoral Neck Fracture

**DOI:** 10.3389/fphys.2021.701467

**Published:** 2021-07-29

**Authors:** Rui Yue, Minghui Yang, Xiaohui Deng, Ping Zhang

**Affiliations:** ^1^Department of Cadre Health Care, Beijing Jishuitan Hospital, Beijing, China; ^2^Department of Traumatic Orthopedics, Beijing Jishuitan Hospital, Beijing, China

**Keywords:** femoral neck fracture, hemiarthroplasty, risk factors, old age, allogeneic red blood cell transfusion

## Abstract

**Objective:**

This study aimed to determine the rate and risk factors of allogeneic red blood cell transfusions (ABT) after hemiarthroplasty (HA) in elderly patients with femoral neck fracture (FNF).

**Methods:**

The subjects of the study were elderly patients (≥65 years old) who were admitted to the geriatric trauma orthopedics ward of Beijing Jishuitan Hospital between March 2018 and June 2019 for HA treatment due to an FNF. The perioperative data were collected retrospectively, and univariate and multivariate stepwise logistic regression analyses were performed to determine the post-operative ABT rate and its risk factors.

**Results:**

There were 445 patients in the study, of whom 177 (39.8%) received ABT after surgery. Multivariate stepwise logistic regression analysis showed that preoperative low hemoglobin (Hb), high intraoperative blood loss (IBL), advanced age, and a low body mass index (BMI) are independent risk factors of ABT after HA in elderly FNF patients.

**Conclusion:**

ABT after HA is a common phenomenon in elderly patients with FNF. Their post-operative ABT needs are related to preoperative low Hb, high IBL, advanced age, and low BMI. Therefore, ABT can be reduced by taking these factors into account. When the same patient had three risk factors (preoperative low hemoglobin, advanced age, and low BMI), the risk of ABT was very high (78.3%). Also, when patients have two risk factors of preoperative low hemoglobin and low BMI, the risk of ABT was also high (80.0%).

## Introduction

Millions of elderly people worldwide suffer from hip fractures each year due to minor accidents or falls (Mitchell et al., 2017), and they are a common reason for the hospitalization of the elderly. Compared with other types of fracture, the incidence of hip fracture in the elderly is high, the mortality rate is high, and patients are prone to losing their independence (Kulshrestha et al., 2019). Treatment methods involve surgery, which can reduce bed time, get patients out of bed as soon as possible, and reduce bed-ridden complications. Thus, it is the approach favored by most physicians.

Femoral neck fracture (FNF) is one type of hip fracture, and hemiarthroplasty (HA) is currently a recognized method for the treatment of FNF. It is considered suitable for elderly displaced FNF with low functional requirements and no acetabular wear (Grosso et al., 2016). Compared with total hip arthroplasty, FNF patients treated with HA have a shorter operation time, lower intraoperative blood loss (IBL), a lower incidence of post-operative dislocation, and a shorter hospital stay, and the financial cost is also lower (Grosso et al., 2016; Liodakis et al., 2016). Nevertheless, HA still has many short- and long-term disadvantages, such as the need for allogeneic red blood cell transfusions (ABT), groin pain caused by acetabular wear, and the potential risk of requiring revision surgery (Grosso et al., 2016; Liodakis et al., 2016). Among them, ABT is the most common, especially for the elderly. However, ABT can have many serious adverse consequences (Zhou et al., 2017; Smeets et al., 2018), including an acute hemolytic transfusion reaction, blood-borne infections (such as human immunodeficiency virus and hepatitis), a prolonged hospital stay, increasing blood resource tension, and patient burden. Unlike other types of surgery, hip surgery often requires a blood transfusion due to certain characteristics, e.g., being a major trauma, involving substantial perioperative blood loss, the fact that elderly patients often have complex basic diseases and limited cardiopulmonary function compensation (Zufferey et al., 2010). In addition, since the elderly have poor tolerance to blood transfusion, blood transfusion-related complications are more likely to occur. It is known that different fracture sites (FNF and intertrochanteric fractures) and the choice of different surgical procedures for the same type of fracture will affect the amount of blood loss, which in turn affects the post-operative blood transfusion rate. There are many studies reporting on the risk factors of post-operative blood transfusions after total hip arthroplasty, such as female gender, high American Society of Anesthesiologists (ASA) classification, and low preoperative hemoglobin (Hb) (Yoshihara and Yoneoka, 2014; Huang et al., 2018). However, there are few research reports on ABT after HA in elderly FNF patients. Therefore, this study looks at the predictors of ABT in such patients to help clinicians identify high-risk patients, carry out early intervention, and reduce the rate of post-operative ABT in the future.

## Materials and Methods

### Patients

The study involved 445 patients who were admitted to the elderly trauma orthopedics ward of the hospital with hip fractures between March 2018 and June 2019 and met the selection criteria. The inclusion criteria were as follows: (1) ≥65 years old, (2) FNF caused by a low-energy injury (such as a fall from standing or osteoporosis), (3) HA for FNF, and (4) no history of bleeding diseases such as bone tumors, chronic liver diseases, or blood system diseases. The exclusion criteria were as follows: (1) open soft tissue fractures, (2) multiple fractures, or (3) pathological fractures. All the patients received the standard HA treatment, and so, based on the policy of restricted blood transfusion, red blood cells were transfused only to patients with Hb lower than 80 g/L or Hb lower than 100 g/L, but with unstable vital signs or obvious symptoms (heart rate >100 beats/min, systolic blood pressure <90 mmHg, chest pain, massive bleeding, or extreme weakness). All the patients were treated with low molecular weight heparin anticoagulation during the perioperative period, and tranexamic acid was used during the operation. This study was approved by the Ethics Committee of Beijing Jishuitan Hospital, with the ethics approval number: Ji Lun Ke Shen Zi No. 201807-11. All participants signed informed consent.

### Observation Indicators

Patient demographic data included the following: the patient’s gender, age, body mass index (BMI), comorbid diseases (such as hypertension, diabetes, coronary atherosclerotic heart disease, cerebrovascular disease, and chronic lung disease), whether they were taking antiplatelet drugs (aspirin and/or clopidogrel), ASA classification, international normalized ratio (INR), and Hb before surgery.

Perioperative and post-operative data included the following: time from fracture to surgery, type of anesthesia (general anesthesia or intraspinal anesthesia), IBL, and the duration of surgery. The IBL was estimated from the weight of the surgical sponges and the measurement of the volume of blood collected by the suction canisters.

### Statistical Analysis

Statistical analysis was performed using SPSS 22.0. Quantitative data that obeyed the normal distribution were expressed as mean ± SD ($\bar{x}$ ± s),and an independent sample *t*-test was used for comparison between groups; quantitative data that did not follow a normal distribution were represented by the median (interquartile range) [*M* (P25, P75)], and the rank sum test was used for comparison between groups; the qualitative data was expressed by frequency and percentage, and the χ^2^ test was used for comparison between groups. Single factor analysis was used to evaluate the relationship between different factors and ABT. Then multi-factor stepwise logistic regression was used to control the confounding effect. Predictors were excluded until the *P*-values of all the predictors were less than 0.05.

## Results

### The General Characteristics

The study included 445 patients aged 65–101 years, with an average age of (82.13 ± 6.96) years, and 141 of them were male (31.69%) and 304 female (68.31%).

### The Univariate Analysis

The univariate analysis in Table 1 shows that there was no statistical difference between the two groups in gender, comorbid diseases, INR, ASA classification, time from fracture to surgery, duration of surgery, or whether antiplatelet drugs were taken 1 week before surgery. The preoperative Hb and BMI of the blood transfusion group were significantly lower than those of the non-transfusion group, and the IBL and age were significantly higher than those of the non-transfusion group.

**TABLE 1:** Univariate analysis of ABT after HA in elderly patients with FNF.

Variable	Non-transfusion group (*n* = 268)	Blood transfusion group (*n* = 177)	*t*/*Z*/χ^2^ value	*P*-value
Gender			3.576	0.059
Male	94	47		
Female	174	130		
Age (year)	81.09 ± 6.78	83.72 ± 6.94	–3.979	<0.001
BMI (kg/m^2^)	22.93 ± 3.73	21.11 ± 3.95	4.920	<0.001
Whether to take antiplatelet drugs 1 week before surgery			0.446	0.504
Yes	68	40		
No	200	137		
Hypertension			3.005	0.083
Yes	166	95		
No	102	82		
Diabetes			3.335	0.068
Yes	85	42		
No	183	135		
Atherosclerotic heart disease			0.036	0.849
Yes	72	49		
No	196	128		
Cerebrovascular disease			1.903	0.168
Yes	84	43		
No	184	134		
Chronic lung disease			0.116	0.733
Yes	41	25		
No	227	152		
Preoperative Hb (g/L)	128.80 ± 12.41	113.18 ± 14.69	12.071	<0.001
INR	1.02 ± 0.18	1.02 ± 0.12	–0.058	0.954
Fracture to operation time (days)	3.0 (2.0, 6.0)	3.0 (2.0, 6.0)	–0.284	0.777
ASA classification			2.670	0.102
<III classification	94	49		
≥III classification	174	128		
Anesthesia method			5.817	0.016
General anesthesia	5	11		
Intravertebral anesthesia	263	166		
Operation duration (min)	64.68 ± 17.65	62.85 ± 18.42	1.050	0.294
IBL (ml)	190.75 ± 82.32	227.12 ± 93.38	–4.322	<0.001
ABT quantity (ml)	–	467.80 ± 237.25		

*INR, international normalized ratio.*

### Multivariate Stepwise Logistic Regression Analysis

Multivariate stepwise logistic regression analysis showed that low preoperative Hb, high IBL, advanced age, and low BMI were independent risk factors for ABT after HA in elderly FNF patients. Among them, the Wald value of preoperative Hb was the largest, indicating that low Hb before surgery has the greatest impact on ABT after surgery (see Table 2).

**TABLE 2:** Multivariate stepwise logistic regression analysis of ABT after HA in elderly patients with FNF.

Risk factors	OR	95% CI	Wald value	*P*-value
Age (year)	1.056	1.020−1.094	9.430	0.002
IBL (mL)	1.007	1.004−1.009	22.816	<0.001
Preoperative Hb (g/L)	0.913	0.894−0.933	72.159	<0.001
BMI (kg/m^2^)	0.916	0.860−0.975	7.585	0.006

### The Multivariate Analysis

As shown in Table 3, the same patient may have two or three risk factors at the same time. We analyzed the number and proportion of ABT in patients with combined risk factors, and found that when the same patient had three risk factors (preoperative low hemoglobin, advanced age, and low BMI), the risk of ABT was very high (78.3%). Also, when patients have two risk factors of preoperative low hemoglobin and low BMI, the risk of ABT was also high (80.0%).

**TABLE 3:** The multivariate analysis.

Risk factors	Preoperative low hemoglobin	Advanced age	Low body mass index	Patients with risk factors	Patients with risk factors and ABT (%)	Patients with risk factors but no ABT (%)	*P*
Patients with three risk factors	Yes	Yes	Yes	23	18 (78.3)	5 (21.7)	<0.05
Patients with two risk factors	Yes	Yes	No	118	77 (65.3)	41 (34.7)	<0.05
	Yes	No	Yes	30	24 (80.0)	6 (20.0)	<0.05
	No	Yes	Yes	59	36 (61.0)	23 (39.0)	<0.05
Patients with one risk factors	Yes	No	No	171	104 (60.8)	67 (39.2)	<0.05
	No	Yes	No	304	129 (42.4)	175 (57.6)	<0.05
	No	No	Yes	70	44 (62.9)	26 (37.1)	<0.05

## Discussion

A total of 445 patients were included in the study, and the outcomes showed that preoperative low Hb, high IBL, advanced age, and a low BMI were independent risk factors of ABT after HA in elderly FNF patients.

Regarding the blood transfusion rate after hip fracture surgery, there have been different reports in the domestic and foreign literature. This may be related to the age, fracture type, comorbid diseases, or blood transfusion policy of the subjects in each study. Arshi et al. (2020) studied 8,416 elderly hip fracture patients over 65 years of age and found that 28.3% of them were transfused after surgery. Many previous studies have concluded that compared with FNF, the risk of blood transfusion after femoral intertrochanteric fracture is increased (Martinsen et al., 2016; Arshi et al., 2020). This study did not include patients with femoral intertrochanteric fractures, but the post-operative ABT was even higher. This may be because this hospital is a large-scale upper first-class general hospital with orthopedics and burns as the key department, so some of the patients admitted were referred from primary hospitals. The patients were older, had many complicated diseases and a relatively severe illness, which also resulted in the relatively high post-operative ABT rate in this study. In the study of Wang et al. (2020), elderly patients with HA after FNF were also chosen as the research objects, but they found that the post-operative blood transfusion rate was only 13.9%, which was significantly lower than the 39.8% in this study. The reasons for this may be that in the Wang et al. (2020) study, the average age of the blood transfusion group was 78.95 ± 5.26 years old, and that of the non-transfusion group was 80.82 ± 5.23 years old, while in this study, the blood transfusion group was 83.72 ± 6.94 years old, and the non-transfusion group was 81.09 ± 6.78. It can be seen that the patients in this study were older, and advanced age in itself is one of the risk factors for post-operative ABT.

At present, there is no clear regulation on the standard of blood transfusion for elderly patients with hip fracture (Zhu et al., 2019). Carson et al. (2016) systematically reviewed the results of 31 studies (involving 12,587 patients) and suggested that the restrictive blood transfusion strategy of controlling the blood transfusion standard at Hb 7–8 g/dL can reduce the ABT rate by 43%. It was also found that it will not increase the mortality, complication rate, and readmission rate within 30 days after surgery, nor will it affect the recovery of patients after surgery. The research of Xie X. H. et al. (2019) shows that for elderly patients undergoing surgical treatment of hip fractures, restricted blood transfusion is safe and effective, does not affect the prognosis of patients, and is significantly better than unrestricted blood transfusion in terms of adverse reactions after blood transfusion and blood saving. Based on the above findings, this study adopted a restrictive blood transfusion strategy.

Multivariate stepwise logistic regression analysis showed that low preoperative Hb, high IBL, advanced age, and low BMI were independent risk factors for ABT in elderly FNF patients after HA. In this study, the preoperative Hb of the blood transfusion group was 113.18 ± 14.69 g/L, which was significantly lower than the preoperative Hb 128.80 ± 12.41 g/L of the non-transfusion group (*P* < 0.05). This result is consistent with many previous studies (Yan et al., 2015; Arshi et al., 2020; Wang et al., 2020). Adunsky et al. (2003) found that patients with preoperative Hb lower than 120 g/L have a fivefold increase in the risk of post-operative blood transfusion. It was speculated that the reason may be due to the poor immune response and compensatory ability of patients with low Hb before surgery when faced with stresses such as surgery and blood loss. Shokoohi et al. (2012) further reported that for every 1 g/dL increase in Hb on admission, the chances of a patient needing a blood transfusion decreased by about 49%.

There are many reasons for preoperative anemia: (Zhou et al., 2017) (1) acute and chronic hemorrhagic anemia, the former caused by fractures and the latter by bleeding from digestive ulcers, intestinal polyps, or hemorrhoids; (2) nutritional anemia caused by the lack of hematopoietic materials, iron deficiency anemia being the most common, and megaloblastic anemia, which is rare, caused by a lack of folic acid and vitamin B; (3) anemia of chronic disease, characterized by disorders of iron metabolism, which occur in the course of some chronic diseases, and which are common in anemia combined with chronic infection, inflammation, and tumor; and (4) other anemia that may involve a variety of complex pathogenic mechanisms and comorbidities. The elderly are more likely to have preoperative anemia due to multiple diseases, reduced absorption, the utilization of hematopoietic materials, and reduced hematopoietic reserves. Yoon et al. (2019) believed that intravenous iron supplementation with restrictive blood transfusion is safe and effective for elderly patients with hip fractures.

This study also found that IBL is an independent risk factor for ABT. The IBL of the transfusion group was 227.12 ± 93.38 ml, which was significantly higher than that of the non-transfusion group (190.75 ± 82.32 ml; *P* < 0.05), which is consistent with the results of Wang et al. (2020). Under normal circumstances, the amount of IBL increases the risk of blood transfusion. Xie J. et al. (2019) believed that intraoperative use of tranexamic acid can reduce intraoperative bleeding, reduce post-operative ABT, and will not increase the risk of post-operative thromboembolic events or other adverse events. Tranexamic acid was used with all the patients in this study.

Consistent with previous studies (Yan et al., 2015; Arshi et al., 2020), we found that advanced age is an independent risk factor for ABT in elderly FNF patients after HA. This may be related to the lower baseline Hb of older people. In addition, elderly patients are more likely to experience unstable vital signs and acute blood-loss-related symptoms after surgery due to reduced organ function and a weak compensatory ability to withstand surgical stress, thus increasing the demand for ABT.

This research shows that a lower BMI is a risk factor for post-operative ABT, similar to previous experimental results (Frisch et al., 2016; Akinleye et al., 2018; Arshi et al., 2020). Frisch et al. (2016) studied more than 2,300 patients and found that after total hip and knee replacement, patients with a high BMI had a lower blood transfusion rate. The author believes that the protective effect of the BMI on the risk of blood transfusion may be related to the increase in overall blood volume with the increase in the BMI. Although obese patients may lose more blood due to larger incisions during surgery, compared with patients with lower BMIs, their estimated blood loss during a particular surgery may have a lower proportion of total blood volume (Frisch et al., 2016). In addition, a lower BMI may also be an indicator of malnutrition in the elderly.

Unlike the study of Yan et al. (2015), our study found that whether antiplatelet drugs were taken 1 week before surgery or not did not affect ABT in FNF patients after HA. The experiment of Abdulhamid (2020) included 325 FNF patients, of which 163 had long-term use of antiplatelet drugs, while 162 had not used antiplatelet drugs. It was found that there was no statistical difference between the two groups in terms of IBL or post-operative blood transfusion requirements. In this experiment, many elderly patients with cardiovascular and cerebrovascular diseases had a high risk of stopping antiplatelet drugs, so antiplatelet drugs were not stopped during the perioperative period.

Previous studies reported that blood transfusion increased the incidence of complications and led to blood shortages, prolonged hospital stays, and increased hospital costs (Dai et al., 2020; Shin et al., 2020). For elderly FNF patients undergoing HA, these problems cannot be ignored. In addition, for elderly patients with potential risk factors, it is necessary to improve blood management. It is currently believed that through risk assessment, careful surgical planning, and the optimization of preoperative evaluation, the need for blood transfusions during the perioperative period of elderly patients undergoing major surgery can be reduced (Clevenger et al., 2015).

We did multivariate analysis of independent risk factors and got interesting results. We found that when the same patient had three risk factors (preoperative low hemoglobin, advanced age, and low BMI), the risk of ABT was very high (78.3%). Also, when patients have two risk factors of preoperative low hemoglobin and low BMI, the risk of ABT was also high (80.0%). This result showed that compared with a single risk factor, we need to be more alert to the risk of ABT when preoperative low hemoglobin, advanced age and low BMI appeared in the same patients or preoperative low hemoglobin and low BMI appeared in the same patients.

This study had several limitations. Firstly, this study was a retrospective trial, not a randomized controlled trial. Secondly, the sample size was limited and will inevitably be affected by inherent data, so another trial with a larger sample size is still needed. Thirdly, all the data in this study came from the same hospital. Therefore, it is necessary to conduct a multi-center study to validate the results.

## Conclusion

In conclusion, ABT after HA is a common phenomenon in elderly patients with FNF. Their post-operative ABT needs are related to preoperative low Hb, high IBL, advanced age, and low BMI. Therefore, ABT can be reduced by addressing these factors. At the same time, we need to be more alert to the combination of risk factors.

## Data Availability Statement

The original contributions presented in the study are included in the article/supplementary material, further inquiries can be directed to the corresponding author/s.

## Ethics Statement

The studies involving human participants were reviewed and approved by the Beijing Jishuitan Hospital. The patients/participants provided their written informed consent to participate in this study.

## Author Contributions

RY and PZ: conception and design of the research, analysis and interpretation of the data, and critical revision of the manuscript for intellectual content. RY and MY: acquisition of the data. PZ: statistical analysis. RY, XD, MY, and PZ: writing of the manuscript. All authors contributed to the article and approved the submitted version.

## Conflict of Interest

The authors declare that the research was conducted in the absence of any commercial or financial relationships that could be construed as a potential conflict of interest.

## Publisher’s Note

All claims expressed in this article are solely those of the authors and do not necessarily represent those of their affiliated organizations, or those of the publisher, the editors and the reviewers. Any product that may be evaluated in this article, or claim that may be made by its manufacturer, is not guaranteed or endorsed by the publisher.

## Abbreviations

ABT: allogeneic red blood cell transfusions
FNF: femoral neck fracture
HA: hemiarthroplasty
Hb: hemoglobin
INR: international normalized ratio
IBL: intraoperative blood loss
BMI: body mass index
ASA: American Society of Anesthesiologists.
